# Human Thalamic Somatosensory Nucleus (Ventral Caudal, Vc) as a Locus for Stimulation by INPUTS from Tactile, Noxious and Thermal Sensors on an Active Prosthesis

**DOI:** 10.3390/s17061197

**Published:** 2017-05-24

**Authors:** Jui Hong Chien, Anna Korzeniewska, Luana Colloca, Claudia Campbell, Patrick Dougherty, Frederick Lenz

**Affiliations:** 1 Department of Neurosurgery, Johns Hopkins University, Baltimore, MD 21287, USA; jchien7@jhmi.edu or cjh425@gmail.com; 2 Departments of Neurology and Cognitive Science, Johns Hopkins University, Baltimore, MD 21287, USA; akorzen@jhmi.edu; 3 Department of Pain Translational Symptom Science, School of Nursing, and Department of Anesthesiology, School of Medicine, University of Maryland, Baltimore, MD 20742, USA; colloca@son.umaryland.edu; 4 Department of Psychiatry and Behavioral Sciences, Johns Hopkins University, Baltimore, MD 21287, USA; ccampb41@jhmi.edu; 5 Department of Anesthesiology and Critical Care Medicine, M.D. Anderson Hospital, Houston, TX 77054, USA; pdougherty@mdanderson.org

**Keywords:** sensor, active prosthesis, thalamus, mechanoreception, nociception, neuron, microstimulation

## Abstract

The forebrain somatic sensory locus for input from sensors on the surface of an active prosthesis is an important component of the Brain Machine Interface. We now review the neuronal responses to controlled cutaneous stimuli and the sensations produced by Threshold Stimulation at Microampere current levels (TMIS) in such a locus, the human thalamic Ventral Caudal nucleus (Vc). The responses of these neurons to tactile stimuli mirror those for the corresponding class of tactile mechanoreceptor fiber in the peripheral nerve, and TMIS can evoke sensations like those produced by the stimuli that optimally activate each class. These neuronal responses show a somatotopic arrangement from lateral to medial in the sequence: leg, arm, face and intraoral structures. TMIS evoked sensations show a much more detailed organization into anterior posteriorly oriented rods, approximately 300 microns diameter, that represent smaller parts of the body, such as parts of individual digits. Neurons responding to painful and thermal stimuli are most dense around the posterior inferior border of Vc, and TMIS evoked pain sensations occur in one of two patterns: (i) pain evoked regardless of the frequency or number of spikes in a burst of TMIS; and (ii) the description and intensity of the sensation changes with increasing frequencies and numbers. In patients with major injuries leading to loss of somatic sensory input, TMIS often evokes sensations in the representation of parts of the body with loss of sensory input, e.g., the phantom after amputation. Some patients with these injuries have ongoing pain and pain evoked by TMIS of the representation in those parts of the body. Therefore, thalamic TMIS may produce useful patterned somatotopic feedback to the CNS from sensors on an active prosthesis that is sometimes complicated by TMIS evoked pain in the representation of those parts of the body.

## 1. Introduction

Cutaneous receptors and sensations play an important role in manual tasks and behaviors that are significant components of human behavior. Tactile cutaneous mechanoreceptors are crucial for exploratory behaviors, such as active touch [1,2], and for fine motor tasks such as typing, manipulating tools or playing a musical instrument [3,4]. Receptors signaling the presence of thermal and painful stimuli are critical for detection and identification of potentially injurious stimuli leading to escape from the stimulus and avoidance of future injuries [5,6,7,8]. Patients with amputations are unable to carry out these behaviors but could regain these abilities with the aid of an active prosthesis. Input of the type produced by cutaneous receptors on active prostheses upon sensory neural structures could improve sensory and motor function, and the functionality of active prosthesis in patients with amputations [9,10]. 

This feedback could encode the tactile or injurious characteristics of an object to be manipulated, and so identify the object, prevent injury, and verify that the movement is being carried out as instructed by the amputee [11]. In the control of hand movements, the signals from cutaneous sensors can be compared with the expected signals as predicted from the motor commands from the amputee to the prosthesis. The motor commands can then be updated as the amputee adapts to the difference between the expected and actual signals arising from the prosthesis [11]. Some neural signals throughout the movement may constitute an efference copy [12,13], which may explain the neuronal activity in the Vc (Ventral Caudal) complex and motor thalamus that occurs during “phantom” movements long after an amputation [14,15]. These neural signals might facilitate learning during training of the amputee to carry out movements with the prosthesis. 

The sensory loci to be stimulated with the signal generated by a sensor on the prosthesis may include nervous structures, such as the peripheral nerves [16,17], spinal cord [18], thalamus [19], and cortex [19,20]. Central nervous system loci that could be stimulated effectively by input from a cutaneous sensor may be identified by their physiologic characteristics, which may predict the likelihood that stimulation at that site will be able to provide useful somatic sensations. 

These features of loci should include: First the presence of cells that respond to tactile, thermal and painful stimuli, and stimulation evoked sensations like those produced by the stimuli that activate mechanoreceptors, thermoceptors or nociceptors. This property will demonstrate that a locus receives sensory stimuli encoded in the periphery, which are then transmitted to cortical structures where they may reach consciousness as sensations, or may modify ongoing movements without reaching consciousness. Second, the location of such neurons and evoked sensations should be segmented by modality and somatotopy, and reflect the density and type of receptor by location on the body [11]. These properties will demonstrate units of isorepresentation, so that Threshold Stimulation at Microampere current levels (TMIS) can produce discrete sensations or modify movements of isolated parts of the body. Third, TMIS presented in a pattern should produce the natural sensation, which can be graded by the parameters of stimulation [10,19]. Finally, the somatotopy and modality of the sensations evoked by stimulation of the sensory locus by the signal from the sensor should be comparable before and after a major injury, such as an amputation. 

Vc can be promoted as a locus by responses to peripheral stimuli and the sensations evoked produced by TMIS. Vc is highly segmented by these responses and sensations into units of isorepresentation for modality and somatotopy. Thalamic TMIS evoked sensations are often natural and so can be interpreted in terms of the patient’s previous experience without prolonged training. Units of isorepresentation are found at predictable locations in a compact nucleus across patients including those with major injuries, which simplify the surgical approach to implantation of the electrode for chronic thalamic TMIS. In addition this nucleus can be approached by minimally invasive procedures whereas the corresponding cortical locus covers a large area deep in the central sulcus [21]. Therefore, implantation of cortical electrodes requires a full craniotomy with additional invasive steps to place electrodes in the central sulcus, or several stereotactic placements, each adding to the risk of hemorrhage For these reasons, Vc is a good candidate to be the locus for input from tactile, thermal or harm signaling sensors based upon physiology and surgical approaches. 

**Figure 1:**
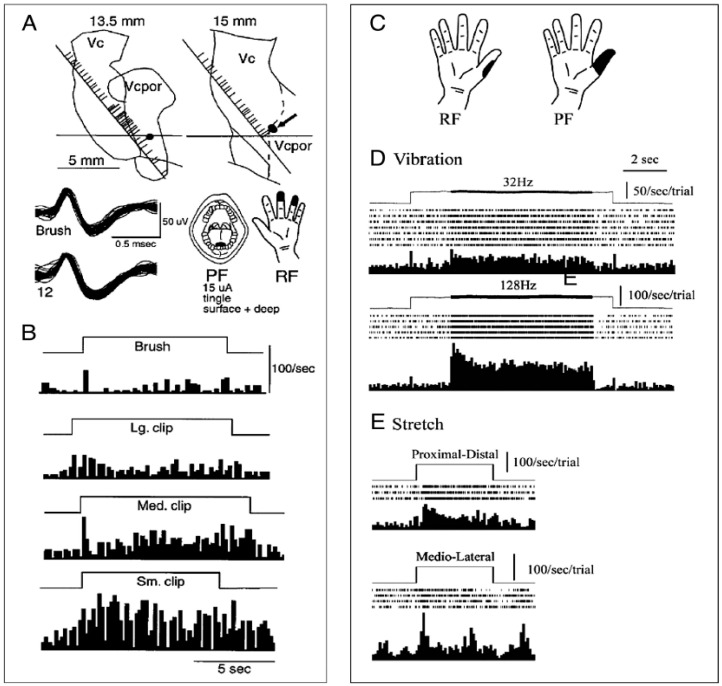
(**A**) Activity of a cell in Ventral caudal (Vc), responding to painful mechanical stimuli, and thermal stimuli (not shown). Location of the cell (arrow) is shown in relation to the positions of trajectories, nuclear boundaries (Ventral caudal portae - Vcpor), and other recorded cells. The ACPC line is indicated by the horizontal line and the trajectories are shown by the oblique lines (left, anterior; up, dorsal). Nuclear location was estimated from the position of the ACPC line. Lateral location of the trajectories (in mm) is indicated above each map. Trajectories have been shifted along the ACPC line until the most posterior cell with a cutaneous Receptive Field (RF) is aligned with the posterior border of Vc. The locations of cells are indicated by ticks to the right of each trajectory. Cells with cutaneous RFs are indicated by long ticks, those without definable RFs by short ticks. Filled circles attached to the long ticks indicate that somatic sensory testing was carried out. The scale is as indicated. The shape of action potentials is recorded at the beginning of the recording from this cell (upper trace) and at the end of the recording (lower trace). Action potential discrimination was carried out by level detectors for different phases of the action potential. The RF and PF (projected field) for the natural, surface and deep, non-painful, tingling sensation evoked by TMIS at the recording site (threshold, 15 μA) are also shown. (**B**) Response to the camel hair brush (BR Pain VAS, 0), Large Clip (VAS, ~2), MC (VAS, ~5), and Small Clip (VAS, ~7). Adapted from [41] with permission. (**C**–**E**) Activity of a cell in Vc core of a patient with ET (different from 1A,B) which showed characteristics like those of a Pacinian fiber (PC). (**C**) Figurines of the RF and PF. (**E**) Response to step indentation and vibration at 32–128 Hz as measured by the Chubbuck stimulator (as labeled). (**D**) Skin stretch, Proximal Distal and Medial Lateral directions as indicated. Adapted with permission from [38].

We now review studies of high impedance microelectrode recordings from individual neurons and TMIS evoked sensations to map nuclear boundaries in patients during awake, stereotactic, thalamic surgeries. The results of these studies will be considered to demonstrate the utility of Vc as a locus for signals from cutaneous sensors. The nuclei that are explored include Vc complex composed of Vc core, Vc portae (Vcpor, posterior to core, Figure 1A), Pulvinar oral (PLo, further posterior), and Vc parvocellular (Vcpc, inferior) [21,22]. During the mapping of Vc complex before thalamic surgical procedures for the treatment of chronic pain, the function and organization of Vc is studied in detail. During thalamic surgery in patients with Movement Disorders, Vc is studied to predict the borders of the thalamic nuclei which are related to movement including ventral intermediate (Vim) as well as the ventral oral posterior (Vop), and which are located anterior to Vc [22,23,24]. During surgery for movement disorders, these nuclei may be lesioned (thalamotomy) [25], or deep brain stimulating (DBS) electrodes may be implanted [25,26]. In addition, DBS electrodes may be implanted in Vc to treat intractable chronic pain due to nervous system injury [27,28,29]. Therefore, Vc is thoroughly explored in patients with Movement Disorders and Chronic Pain, and the results of these explorations are used to establish the target for direct surgical lesioning or placement of DBS electrodes [25]. 

In preparation for the exploration and surgery, the location of the nucleus to be lesioned or stimulated is estimated by MRI and CT imaging. Microelectrode studies are then used to confirm the location of nuclei both by recordings of neuronal activity and by TMIS. These microelectrode studies are a unique window into the characteristics of Vc complex in patients with Essential Tremor (ET) who do not demonstrate abnormalities of the cutaneous sensory system [30,31], as well as in patients with nervous system injury and other Movement Disorders [30,31]. 

This review will focus on the thalamic neuronal activity in the Vc complex and on TMIS activity related to tactile and thermal pain sensations recorded during explorations to target placement of lesions of Deep Brain Stimulating (DBS) electrodes [25,32]. Technical arrangements and details regarding the sensors, signal conditioning, and electrodes in the brain are beyond the scope of this paper. Some of the results reported here have previously been published [33,34,35,36,37,38,39,40,41]. The studies from our lab described here were reviewed and approved yearly by the Institutional Review Boards of the Johns Hopkins University School of Medicine. All patients signed the approved informed consent for participation in these studies.

## 2. Neuronal Responses and Microstimulation Evoked Sensation Related to Cutaneous Stimuli

Human thalamic responses to quantitative cutaneous mechanical stimuli have been studied by a technique similar to those employed in basic studies [38,42,43]. Four stimuli were applied during recording from each neuron; each stimulus was chosen to evoke activity selectively in one of the four mechanoreceptor types (the optimal stimulus for that type). Figure 1C,D shows an example of a thalamic neuron with Pacinian Corpuscle (PC) like behavior based upon a response to mechanical stimuli like that of PC mechanoreceptors, and with a Receptive Field (RF) on the dorsal surface of the thumb (Figure 1C). The firing rate for this neuron showed a transient peak at the time of skin indentation (0.5 mm) and at the reversal of indentation for the 32 and 128 Hz vibration stimuli. Following the indentation the response to vibration (0.1 mm peak to peak) was significantly greater for the 128 Hz (the optimal stimulus for PC) than to the 32 Hz vibration (Figure 1D), and greater than for stretch, in both the medial lateral and the proximal distal direction (Figure 1E). This cell also responded to edge, and percentage entrainment by vibration was significantly greater for 128 Hz than for 32 Hz. We found that seven neurons had an optimal response to 128 Hz. 

TMIS in Vc evoked a deep vibration sensation at one out of seven sites, and a deep, touch, electric warm sensation at another site. This is different from the sensation of vibration or buzzing that is commonly evoked by intraneural TMIS, which is thought to produce impulses in single fibers, in this case a PC mechanoreceptor fiber (discussed in reference [6]).

Seven rapidly adapting like (RA) neurons showed a response to vibratory stimuli at 32 or 64 Hz, which was significantly greater than the response to any other optimal stimulus. TMIS at or adjacent to the thalamic recording site evoked deep vibration sensations at four sites while a surface, sharp, moving sensation was reported at one site. TMIS of RA fibers in peripheral nerves innervating the hand produces a sensation of tapping or flutter [6]. 

Among slowly adapting type 1 (SA1) like neurons, two had a combined tonic and phasic response to edge stimulation, which was significantly greater than for any other stimulus. One neuron had a greater response to edge in one orientation, which was significantly greater than the response to any other stimulus except both 128 and 32 Hz vibration stimuli. Therefore, input arising from SA1 mechanoreceptors (optimal stimulus-edge) showed evidence of convergence with that from PC or RA or both, as indicated by responses to 128 or 32 Hz vibration [44]. TMIS produced an unnatural, surface and deep vibration sensation in a part of the body (Projected Field, PF), which overlapped with the Receptive Field (RF) in two neurons, and an electric current sensation for one neuron. Intraneural TIMS of SA1 fibers produced a pressure or pulling sensation [6].

Three slowly adapting type 2 (SA2) like neurons showed an increase in firing response to stretch in one or both directions. One of these had a response to 32 Hz that was not significantly different from that to stretch in either direction, so there may be convergence with input from SA1 or RA2 mechanoreceptors upon this neuron. TMIS at the site of these neurons evoked vibratory sensations, which overlapped with the RF. TMIS of SA2 fibers in the peripheral nerve evoked “no distinct and consistent quality of sensation” [6]. 

There are some mismatches between peripheral nerve versus thalamic TMIS evoked sensations, and between the mechanoreceptor fibers versus the corresponding Vc neuronal responses to mechanical stimuli. In the first case, the absence of distinct TMIS evoked sensation for SA2 fibers suggests that stimulation of SA2 like neurons will not likely produce a distinct sensation. The indistinct sensations could also reflect the convergence of input arising from other mechanoreceptors on SA2 like neurons. Vibration sensations may be more common because the dense neuropil of Vc contains more RA like and PC like neurons than SA like neurons, including 14/20 mechanoreceptor like neurons plus axons and dendrites. Therefore, stimulation in Vc is more likely on average to activate these elements and produce sensations of vibration (in this series 40% of TMIS sites). In addition, stimulus trains of TMIS may not correspond to the patterns produced by optimal stimulation of a mechanoreceptor afferent fibers or mechanoreceptor like neurons; therefore, natural, recognizable or distinct sensations may not be evoked [6]. 

The activity of most Vc neurons responding to optimal mechanical stimuli is similar to that of the corresponding peripheral mechanoreceptors [42,43], although the neurons with SA1 and SA2 like activity often showed mismatches consistent with the convergence with input from other mechanoreceptors. The responses of neurons in human Vc were also similar to neurons in the Ventral Posterior nucleus (VP, corresponding to human Vc [21]) of anesthetized marmosets and raccoons [21,45,46], while all may reflect the distribution of mechanoreceptors in different parts of the body [38,39,43]. Overall, these results demonstrate surprising fidelity of activity of the mechanoreceptor like Vc neurons to that of mechanoreceptor fibers, in spite of the axonal spans, and the (limited) circuitry of the dorsal root ganglion and dorsal column nuclei between them.

However, it was not clear how these responses were matched to the sensations evoked by TMIS adjacent to the recording site, except in the case of RA like neurons. For these neurons TMIS adjacent to the recording site produced the expected vibration sensation at the majority of sites. In spite of the mismatch between neuronal responses and sensations evoked by TMIS in Vc overall, the sensations evoked by optimal stimuli can be produced by many stimulation sites in Vc [37]. 

In human thalamic Vc TMIS produced sensations in the mechanical and movement category (33/128 TMIS sites) included: “pressure” (17 cases), “sharp” (five cases), “vibration” (40 cases), and “movement across cutaneous structures or deep structures” (27 cases) [37]. The PFs at these 128 sites were located on mucosal surfaces in 29 cases and on hairy skin of the upper extremity in six cases and so may reflect the sensation produced by activation of different mechanoreceptor afferent fiber populations and types on these surfaces.

Among 122 TMIS sites where mechanical or movement descriptors were endorsed, the RF at the nearest recording site was overlapped (matched) with the PF at 54% of the sites. The proportion of matches was not different between different mechanical and movement descriptors. The descriptors for TMIS evoked sensations were more frequently described as “natural” at sites with a match (52%) than at those with a mismatch (31% of ET cases [34]). The frequent mismatch sites may have resulted from activation of axons that stream through thalamic nuclei and are afferent to or efferent from the Vc complex. These fibers of passage often signal different RFs and peripheral stimuli than the adjacent neuronal somata. TMIS evoked “touch” and “vibration” descriptors were endorsed more frequently than “pressure” in facial PFs, while “pressure” was evoked more commonly than “vibration” at intraoral PFs [37]. These differences in PF location may reflect differences in the distribution of mechanoreceptors and may result from and influence the sensory functions in different parts of the body. 

## 3. Thalamic Vc Activity Related to Thermal or Painful Stimuli

There were also neural responses to thermal and painful stimuli (Figure 1A,B), which are shown for a neuron in Vc core with a response graded from the non-painful into the painful range for thermal (not shown) and mechanical stimuli (Figure 1A,B, Wide Dynamic Range (WDR) neuron) [41,47,48]. This cell had significant increases in firing across the series from Brush (camel hair brush, BR) to Small Clip (SC) (Figure 1 Legend), and from non-painful to painful heat (not shown). 

We also described a response pattern defined as Multi Receptive (MR) for cells that showed responses to mechanical stimuli from the non-painful into the painful range that were not graded with intensity. MR neurons were often included in the WDR category in studies of monkeys [48,49,50] (cf. [51]). Activity arising from nociceptors is transmitted to the thalamus largely through the spinothalamic tract (STT). However, the dorsal column and the postsynaptic dorsal column pathways may also transmit these signals to the thalamus [7,48], and neurons with similar properties have been reported in the dorsal column nuclei of anesthetized primates [7,52]. 

## 4. Segmentation by Modality and Somatotopy

Discrete anatomic structures in the thalamic Vc core include somatotopic parasagittal regions with the tongue represented medially and the leg represented laterally in monkeys [7,53,54] and humans [35]. Within this representation of parts of the body is a more fine grained segmentation by different RFs and input from different mechanoreceptors. Anatomic structures, like digits, are arranged in concave medial lamellae within the representation of larger parts of the body, like the hand (see Section 5).

**Figure 2:**
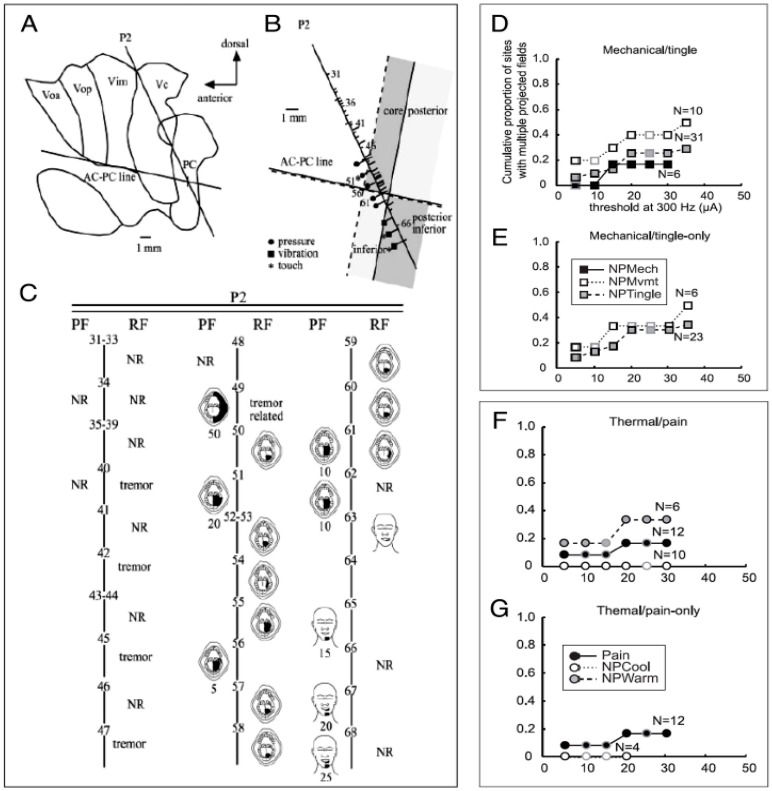
Map of RF and PFs along trajectories in the Vc core in a single patient (**A**) Positions of the trajectories relative to nuclear boundaries as estimated radiologically from the location of the anterior commissure-posterior commissure (AC-PC) line [25]. The AC-PC line is indicated by the approximately horizontal solid line; the trajectory is shown by the oblique line labeled P2. Scale is as indicated. (**B**) Location of recordings and stimulation sites along a trajectory and the ventral border of the core of Vc (approximately horizontal dashed line). The approximately dashed and solid approximately vertical lines are the posterior and anterior borders of the core of Vc, as determined physiologically. Cells are indicated by ticks to the right of the trajectory and those with an RF are indicated by long ticks; those without by short ticks. The TMIS evoked sensations are indicated by the shape at the end of the long ticks to the left. Scale is as indicated. (**C**) Each site at which a neuron was recorded or TMIS delivered are indicated by the same number in B and C which shows the PF and RF by site number. TMIS levels (in microamps) are indicated below the PF, NR, no response. (**D**–**G**) Shows the proportion of sites at which TMIS evoked sensations in more than one part of the body. For the hand, one part would include the dorsal surface and ventral surface of: each digit separately, the hypothenar eminence, thenar eminence and palm (plus corresponding PFs on the dorsal surface). (**D**,**E**) Results for mechanical and tingle sensations (“mechanical/tingle only” indicates sites where Thermal Pain sensations were never evoked). (**F**,**G**) Results for Thermal Pain evoked sensations described with a standard Questionnaire [39]. The insets in **E** and **G** indicate the sensations for each of the symbols, e.g., Black square indicates non-painful (NP) mechanical sensation. Current levels with a gap in sensation evoked by the stepwise increase in current for four sites or more are indicated by a gray perimeter rather than a black perimeter. The number of sites included is indicated to the right of the plot. Plots composed of results from four sites or fewer were excluded. Reproduced with permission: A–C from [37], and D–G from [39].

In addition, anterior-posteriorly oriented rods have been described anatomically within the monkey thalamic principal sensory nucleus (Ventral Posterior, VP) [53,55]. These rods are defined by the terminations of axons originating in the medial lemniscus [56,57], and by neuronal responses to innocuous stimulation [35,53,54,58,59]. The dendrites of thalamic relay neurons run parallel to the rod from which they take their origin [60] and their terminations are aligned with columns in somatosensory cortex (S1) [53] giving rise to the specificity of primary Somatic Sensory cortex (SI) by Somatotopy and Modality [53,55]. These results are consistent with clustering of thalamic neurons along a trajectory by slowly versus rapidly adapting, and touch versus pressure as studied with hand held stimuli [35,53], and clustering by sites at which sensations appropriate for a peripheral mechanoreceptor were produced by TMIS (Figure 2C) [37]. For example, in Figure 2C, there were five consecutive sites at which TMIS evoked pressure (sites 51 to 62) and three at which vibration sensations were evoked (sites 65 to 68).

If there are discrete elements or rods mediating Modality and Place specificity in human Vc, then the proportions of sites with sensations related to more than one descriptor or more than one part of the body (PF) should increase with increasing current of TMIS. However, at some sites within a rod TMIS will cause activation over volumes which are within the rod over a range of increasing current so that there will be plateaus of constant proportions with increasing current. In fact, elements for somatotopy appear as plateaus in plots of TMIS current (at 300 Hz) versus the proportion of sites with PFs at more than one part of the body (Figure 2D,F) or with more than one descriptor (Figure 2E,G). 

The proportion of sites with more than one part of the body often increased from the lowest level at demonstrated plateaus (see Figure 2 Legend) (e.g., 20–30 microamperes in Figure 2D–G). The proportion of sites with more than one part of the body increased again at currents of above 30 microamperes (Figure 2D). At sites where the descriptors for only one modality, such as Mechanical Tingle or Thermal Pain were endorsed, the results were presented for Mechanical Tingle only or Thermal Pain only (Figure 2E,G). The number of these plateaus is remarkable and suggests that the unit of isorepresentation is within the diameter of tissue activated by stimuli of 20 to 30 microamperes, corresponding to a diameter of approximately 300 microns [61]. The number of plateaus over which mechanical tingle sensations was much smaller than those for PFs referred to one part of the body. The results provide strong evidence that elements defined by TMIS are responsible for Modality and Place specificity of non-painful mechanical sensations, and for the intensity coding of somatic sensation produced by TMIS. 

A similar arrangement is found for the Thermal Pain system, although the number of plateaus over which modality stayed constant is much less than for Mechanical Movement sensations. This architecture is consistent with STT terminations which have often been described as “disseminated bursts” in monkey VP, and Vc core of patients following anterolateral cordotomy [55,62,63,64,65]. The “disseminated bursts” define divisions that may be congruent to the position of neurons which respond to painful or thermal stimulation, and to the matrix (staining for calbinden calcium binding protein) which is found between rods in monkey VP [41,49,57,64,66]. The STT also terminates in Vim to a lesser degree, and in the area below and behind Vc where TMIS evokes thermal and pain sensations [67,68,69]. 

**Figure 3:**
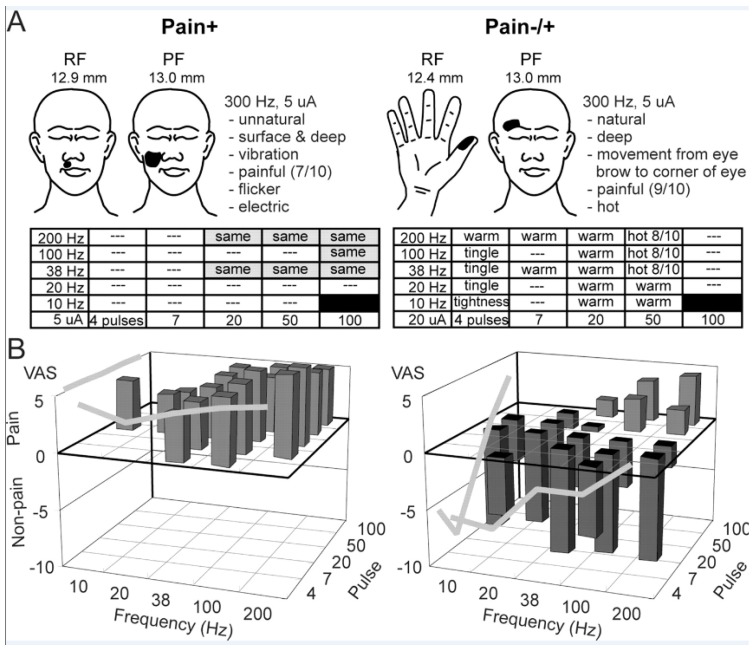
Sensations evoked by TMIS up the burst ascending staircase in patients with movement disorders. (**A left**) site where stimulation at 300 Hz and 5 µA produced pain in the PF and descriptors (top). Pain identical to that evoked by 300 Hz was evoked at sites with shaded rectangles. (**A right**) Sites where tightness, tingle, warm and hot were evoked are indicated along the staircase. Except for a few sites where no sensation was evoked, warmth was evoked at all steps in the ascending staircase from four pulses and 200 Hz to 50 pulses and 20 Hz. At subsequent steps along the staircase, only painful heat was evoked. (**B**) Average VAS ratings across all Pain+ and Pain−/+ sites are shown to the left and right. The light and dark gray bars indicate painful and non-painful TMIS evoked sensations, and gray lines along the outside of the 3D images indicate the average VAS ratings across sites and patients. Reproduced with permission from [70].

Pain sensations were also examined with a burst staircase composed of a train of stimuli of bursts with 4, 7, 20, 50, or 100 pulses, each of which was presented in an ascending series of frequencies from 10, 20, 38, 100 to 200 Hz (Figure 3A). For example, bursts of four pulses were presented at 10, 20, 38, 100 to 200 Hz before bursts of seven pulses were presented at each of these frequencies, and so on. We measured TMIS evoked descriptors and VAS scores and found that PFs remained constant while descriptors and VAS could remain constant or change along the ascending burst staircase [70]. For Tactile sensations, Modality and Place-specific representations were found by PF in the ascending burst staircase. These results suggest that different modalities of cutaneous sensation might be relayed through psychophysically defined Modality and Place specific elements in Vc for both non-Painful Mechanical and Thermal Pain sensations. Although the Thermal Pain sensations are more common in posterior Vc and posterior and inferior to Vc core, they overlap with non-painful mechanical sensations [39,70]. 

## 5. Patterned TMIS Should Encode Discrete Natural Sensations 

The propensity of thalamic relay cells to fire in low threshold calcium spike related bursts following inhibitory events (post-inhibitory bursts) has long been associated with drowsiness and sleep [71,72,73]. More recently, these bursts have been associated with the response to sensory stimuli, including somatic sensory stimuli [40,74,75]. The recovery from membrane hyperpolarization will lead to deinactivation of the T calcium conductance which leads to of a calcium spike crested by a post-inhibitory burst of action potentials [76]. 

Post-inhibitory bursts are associated with a pattern of interspike intervals (ISI) between neuronal spikes, preceded by a long ISI (inhibition), and starting with a short ISI (<6 ms) [36]. The ISIs then become progressively longer so that firing decelerates until the burst ends with an ISI of longer than 16 ms. Single spikes occurring between bursts and in the single spike firing pattern occur at a rate of 10 to 20 Hz (principal event rate). In post inhibitory bursts, the number of action potentials in a burst is correlated with the extent of preburst inhibition. WDR and MR neurons as well as neurons responding only to innocuous stimuli showed clear post-inhibitory bursting, which occurred frequently during the response to multiple mechanical and thermal stimuli [40]. 

The metrics of preburst inhibition were, for the most part, unrelated to the type of neuron and stimulus. WDR and MR cells which also responded to cold stimuli had burst rates in response to cold stimuli that were more rapid than those in response to the other stimuli but were not related to the principal event rate. Overall, bursting was related to the type of neuron but not the stimuli, which suggests that it may be less dependent on inhibition resulting from thalamic afferents than on the properties of thalamic relay neurons and their interaction with cortex in thalamo-cortical assemblies [77,78]. 

The sensory consequences of this bursting activity were examined by applying TMIS with pulse trains composed of bursting patterns by the burst staircase described above. Our studies of TMIS evoked Thermal Pain sensations along the burst staircase reveal two categories of response, one analogue or graded with increasing microstimulation intensity (Pain−/+), and one binary (Pain+). Microstimulation of the Pain+ TMIS sites evoked a relatively intense pain and was described by descriptors. VAS ratings that remained unchanged up the burst staircase and the pain intensity was greater than that at Pain−/+ sites (Figure 3). 

Stimulation of the Pain−/+ TMIS sites evoked pain in PFs, which changed across the burst staircase (Figure 3A, right—tightening to tingle, warm and then painfully hot). Therefore, Pain+ sites seem to reflect activity in an exteroceptive labeled line signaling the detection of noxious external stimuli, which may result in an all or none response. At Pain−/+ TMIS stimulation sites, the descriptors and VAS intensity ratings were consistent with an exteroceptive labeled line for identification of the stimulus and for graded responses to different stimuli. These ratings were correlated to the stimulus evoked firing rates of neurons adjacent to the TMIS site in response to stimuli producing same numerical rating as the TMIS evoked sensation. Therefore, the initial rise in ratings for sensations evoked by thalamic TMIS stimuli at Pain+ versus Pain−/+ is consistent with the shorter dynamic range of thalamic NS cells [64], versus the wide dynamic range of WDR cells. 

Of course, the TMIS evoked sensations are likely related to the activity of multiple cells since stimulation with as little as 5 µA causes activation over a distance of 50 to 100 microns [61]. Cells responding to noxious stimuli are clustered both by location within VP [79], and by location within the spaces between rods which form the compartmentalized, tactile cutaneous representation of VP and Vc [35,41,49,53,57,64,66]. Cells responding to noxious stimuli may be located in clusters that are within or adjacent to the “disseminated burst” terminations of the STT in monkey VP, which contain neurons with common sensory properties [64]. In addition, STT terminations are preferentially located in posterior inferior Vc and in the posteriorly adjacent region [62], as well as the corresponding nuclei in monkeys [7,21,48]. However, the sites where thermal pain or mechanical movement sensations were evoked by TMIS were not apparently clustered or somatotopically arranged by part of the body, perhaps as a result of the activation of adjacent axons [37,68]. 

## 6. Somatotopy of RFs and PFs Following Amputation or Spinal Cord Transection 

Thalamic cutaneous sensory processing in patients with major injuries to the somatic sensory system was studied at the time of surgery to implant DBS electrodes for the treatment of chronic pain, including that resulting from spinal cord transection and amputation. In patients with both these injuries, changes in RFs, PFs and ongoing activity were very similar but were more pronounced in patients with spinal transection. Therefore we will now focus on studies in the latter. 

**Figure 4:**
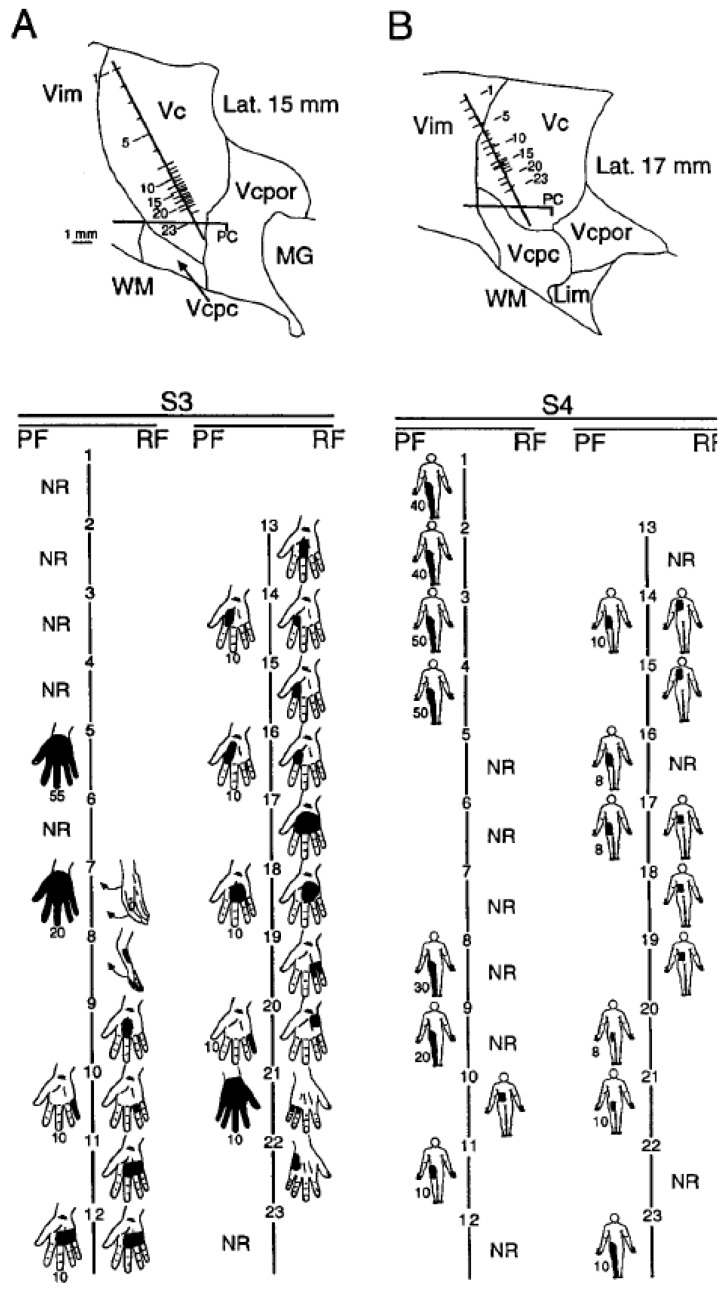
Map of RFs and PFs for trajectories in the region of Vc in a patient with spinal cord transection at Thoracic level 8. Conventions are as described in the Legend for Figure 2. (**A**) Trajectory in 15 mm lateral parasagittal plane (labeled Lat 15 mm) through Vc which represents the arm. (**B**) Trajectory 2 mm lateral to the first (Lat 17 mm). The upper panel shows the position of the trajectory, the AC-PC line, and the recording and stimulation sites. The descriptor tingling was endorsed at all sites. Conventions as in the legend for Figure 2. Reproduced with permission from Figure 1 in [36].

In these studies, it is often difficult to determine the physiological borders of nuclei within the Vc complex, as described in the *Introduction*. On the practical level we defined Vc complex in patients with major injuries as the cellular thalamic region where sensations were produced at less than 25 microamperes (Figure 4 (S3: 7 to 22; S4: 5 to 19)). The Vc complex in patients with such injuries was segmented based on RF and PF locations as follows: (i) “Control” regions which represented structures remote from the part of the body that is anesthetic or absent (e.g., arm in an amputee of the leg, see Figure 4 (S3); (ii) “Insensate” regions and parts of the body at which the patient had partial or complete sensory loss, or an amputation (Figure 4, S4); and (iii) “Tremor” regions in Vc of patients with ET. 

The Vc core was studied in patients with spinal cord transection leading to the type of pain which is mediated by the central nervous system following these injuries (Spinal Central Pain) [36]. Neurons in the Vc complex representation of the “Insensate” part of the body had no RFs (Figure 4B) while many cells had No Response (NR cells), which are indicated to right of the vertical line between PFs and RFs in Figure 4 [36]. Sites at which TMIS produced no sensation are indicated by NR to the left of this line. The detailed representational map of the “Control” area (S3) was similar to the “Tremor” representation (Figure 2A–C), including concave medial lamellae as units of isorepresentation. 

Figure 4B (trajectory S4) shows the representation of rays 3 to 5 (composed of fingers and metacarpals 3 to 5), which are located lateral to the representation of rays 1 and 2 in the somatotopy or homunculus of Vc. Along trajectory S4, the representation of rays 3 to 5 is located anterodorsal (sites 9 to 14) and posteroventral (18 to 22) to the representation of Rays 1 and 2 (sites 14 to 16). This transition from laterally represented to medial and then lateral again for the cells found along trajectories in the parasagittal plane is consistent with the concave medial lamellae which have been described in monkeys [35,53,80] and humans ) [37]. 

The thalamic representation of the body parts next to the “Insensate” representation are increased in comparison to that in “Control” and “Tremor” areas. Figure 4 shows the map for a patient with a complete transection of the spinal cord at the Eighth Thoracic Vertebral level. PFs and RFs showed substantial overlap at sites in the “Control” areas (Figure 4A), whereas in the “Insensate” areas PFs and RFs did not overlap (Figure 4B). The representation of the leg (Figure 4B, sites 9 to 20) shows no leg RFs as a result of the injury, while PFs were referred to the leg as part of the representation of the “Insensate” part of the body. 

In the “Insensate” representation, RFs are located on the chest and abdominal wall above the Eighth Thoracic dermatome, while the PFs to TMIS in the “Insensate” part of the thalamus were found below. Therefore, the activity of thalamic cells representing the part of the body above the injury will lead to sensations in the “Insensate” part of the body. This may include the pathologic patterns of thalamic neuronal activity which involve the “Insensate” thalamic representation in patients with amputation and spinal cord transection [33,36]. These patterns may be consistent with the finding that Pain+ sites in patients with a range of chronic pain diagnoses are more common than in patients with ET. The description, intensity of TMIS evoked pain, and the location of the Pain+ and Pain−/+ sites were not significantly different between patients with chronic pain versus ET [70].

In patients with amputations, neurons with RFs on and adjacent to the stump showed a larger representation [81] than the corresponding part of the body in patients with ET [35,36], which is evidence for a change in the map of somatic afferents from the limb. The PFs show an increased representation of the stump contrasted with that for the same part of the body in controls [37,82], which is evidence for a change in the representation of the arm embedded in thalamic-cortical assemblies [83,84]. 

In patients with a phantom, sensations in that phantom can be evoked by stimulation of sites in Vc core in which stump RFs are found [85]. This latter phenomenon is like that in patients with spinal transection who have persistence of the PFs of the “Insensate” leg (Figure 4B). In the patients with spinal transection or amputation the factors that lead to preservation of the representation of the “Insensate” part of the body are unclear, but may depend upon somatotopic extent in the thalamus or the dimensions of the “insensate” part of the body [86]. 

## 7. Thalamic Function in Primates with Major Injuries to the Somatic Sensory System 

The post-inhibitory bursting that occurs during sleep, drowsiness and the response to sensory stimuli, is also found in patients with major injuries of the somatic sensory system. In patients with spinal transection, the highest burst rates occur in neurons without RFs, and are located in the “Insensate” thalamic representation as identified by TMIS. These cells also have the lowest single spike firing rates during the intervals between bursts [36], which suggests that they are hyperpolarized, perhaps due to loss of tonic excitatory input from the STT [87,88]. Therefore, the effected thalamic cells in patients with spinal transection are dominated by bursting which may be due to membrane hyperpolarization [76]. 

Bursting activity is maximal in the region at and posterior-inferior to Vc core [36], and stimulation in this region evokes the sensation of pain more frequently than in Vc core [68,89,90,91,92]. Furthermore, in the representation of the border of the “Insensate” region in Dorsal Root Entry Zone recordings shows abnormal activity, while lesions relieve the pain and dysesthesias [93,94,95]. These results suggest that increased post-inhibitory bursting in the border of the representation of the “Insensate” region may be correlated with some aspects of the pain and dysesthesias that these patients often experience [96,97,98]. There is an overlap between the areas of chronic pain with those of sensory loss in patients with spinal transection [36,99], which suggests that burst activity may be a result of sensory loss rather than a result of Spinal Central Pain following the injury. 

Individual neurons in both “Insensate” and “Control” thalamic representations showed post-inhibitory bursting and single spike patterns [81]. Overall, cells with burst firing patterns of the post-inhibitory type were more common in patients with spinal transection than in patients with movement disorders, and were more common in the “Insensate” regions than in the “Control” regions. In amputees, post-inhibitory bursts were also found in the “Insensate” representation in the thalamus, particularly in the activity of cells with RFs in the stump area. In these cells, spike rates between bursts (primary event rates) were significantly higher than in other cells in the Vc complex, suggesting that the bursts are not due to hyperpolarization but to the dendritic potentials that lead to burst activity [100,101]. 

The findings regarding burst activity in patients with spinal transection or amputation have been disputed by the results of a study of consecutive patients undergoing DBS implants for the treatment of chronic pain of multiple diagnoses [99]. This report found that the number of bursting cells per distance along the trajectory among patients operated for the treatment of movement disorders was not different from those operated for the treatment of chronic pain. However, this study had significant technical differences from our earlier studies in terms of the diagnoses (multiple chronic pain diagnoses versus Spinal Central Pain and stump/phantom pain), the location of cells studied (Vop, Vim, Vcpor, and Vcpc versus Vc core), and the methods of burst analysis. Our earlier studies carried out postoperative analysis of the parameters of post-inhibitory bursts versus the later study, which measured the incidence of bursting cells per distance along the trajectory, as identified by visual examination of the spike train in the operating room. The bursting activity demonstrated in the earlier studies is likely to represent calcium spike associated bursting in the primary somatic sensory nucleus of patients with Spinal Central Pain [102]. 

Thalamic recordings were also carried out in monkeys with a thoracic anterolateral cordotomy [103,104,105]. Some of these animals showed increased behavioral responses to electrocutaneous stimuli, and so may be a model of central pain following a spinal injury [106]. The most pronounced changes in firing pattern were found in thalamic MR cells (above), which had significantly increased bursts occurring both spontaneously during ongoing activity, and in response to brush or clip stimuli. The changes in bursting behavior were widespread, occurring in the thalamic representation of upper and lower extremities, both ipsilateral and contralateral to the cordotomy. In addition, there appears to be a relationship between burst firing and acute pain in the case of some human thalamic WDR and MR cells, as described above [70,107]. 

LTS bursts are also found in the thalamic representation of the monkey upper extremity and of the representation of the arm and leg ipsilateral to a thoracic anterolateral cordotomy [104]. Pain is not typically experienced in these parts of the body in patients with thoracic spinal cord transection or cordotomy [27,36]. Post inhibitory bursts are increased in frequency during slow wave sleep in all mammals studied [108], including man [109,110]. However, such bursting could cause pain if stimulation in the vicinity of the bursting cellular activity produced the sensation of pain. This finding has been reported in studies of sensations evoked by stimulation of the region of Vc in patients with chronic pain secondary to neural injury [82,111]. 

We have examined the modalities of the sensation produced by TMIS in patients with chronic neuropathic pain due to several diagnoses versus patients with movement disorders [82]. These results showed that patients with chronic pain had more sites at which TMIS evoked pain and fewer at which warm and cold (thermal) sensations were evoked. These results were consistent with the explanation that sites at which warm or cold sensations was evoked by TMIS in patients without chronic pain were relabeled so that pain was evoked by TMIS in patients with chronic neuropathic pain. These results are consistent with the sensations reported in historical studies of the response cortical stimulation cortex in patients with chronic pain in a stump or phantom, and this chronic pain is relieved by a cortical resection [112,113,114]. 

## 8. Implications for Forebrain Loci for TMIS by Signals from Sensors

The features that identify forebrain sites to be stimulated by signals from a cutaneous sensor include segmentation by TMIS evoked modality and somatotopy. The studies reviewed demonstrate that the activity of most Vc neurons responding to mechanical stimuli is similar to the responses of peripheral mechanoreceptors to their optimal stimulus [38,42]. The somatotopic location of these neurons also reflects the density and category of mechanoreceptors by cutaneous site [11]. Although neuronal responses consistent with each mechanoreceptor category were observed, the sensations evoked by TMIS at sites adjacent to these neurons usually did not reflect the sensations produced by the optimal stimulus for that category. In large part, this mismatch is due to fibers of passage which connect with multiple distant neurons and are activated by TMIS at low currents [56,61,78]. In spite of the mismatch TMIS in Vc can evoke sensations that are like those produced by the optimal stimuli, and are often judged to be like stimuli in the natural environment, i.e., natural [37]. TMIS of cortex can also produce a sensations like natural stimuli, as demonstrated by movement sensations in response to stimulation of the secondary somatic sensory cortex [115]. 

Neurons that responded to graded mechanical stimuli with an WDR pattern often responded to cold stimuli, and a few responded to warm stimuli [41]. Another small population in Vc core responded to both cool and innocuous mechanical stimuli [116]. Here again there was a mismatch between neuronal responses and TMIS evoked sensations, but pain and non-pain sensations in the thermal and mechanical modalities were often evoked by TMIS. Therefore, TMIS of the Vc complex of evoke sensations like those produced by tactile, thermal and painful stimuli and could be produced by TMIS transmitting signals generated by a tactile sensor.

## 9. Segmentation within Vc

Discrete structures in the primate somatic sensory thalamus include units of isorepresentation which are parasagittal lamellae with the tongue represented medially and the leg represented laterally in monkeys [53,80,117] and humans [35]. This representation of parts of the body is further segmented into smaller units of isorepresentation by RFs and PFs and by different mechanoreceptor types in monkeys [53,55] and humans [35,107]. TMIS evoked natural innocuous mechanical sensations in human Vc show a pattern for stimulation sites along the trajectories in the parasagittal plane, which are consistent with evidence of terminations of axons from the dorsal column nuclei and the STT, and with detailed mapping studies in monkeys and humans [53,57,107,118]. These results suggest that human thalamic TMIS driven by a cutaneous sensor for control of an active prosthesis could successfully exploit this parcellation to produce sensations like those produced by the optimal stimuli for mechanoreceptors or thermal pain receptors. 

Other features predicting the success of a forebrain locus is that TMIS produces sensations like those evoked by the peripheral stimuli and that graded TMIS can selectively activate units of isorepresentation [10,19]. TMIS evoked sensations over the smallest unit of isorepresentation (e.g., part of a digit) commonly remained constant at several steps of increasing current, a Plateau (Figure 2D–G) [20,107]. Plateaus like this were not observed for the proportion of sites at which more than one descriptor is endorsed (e.g., touch, pressure, vibration, movement descriptors within mechanical movement category). This suggests that the termination of the human Medial Lemniscus is arranged into the anatomic units with Modality specificity. These results are strong evidence that the units of isorepresentation defined by TMIS are responsible for Modality- and Place-specificity of non-painful mechanical sensations, and that graded TMIS can selectively activate these units. These properties suggest that stimulation by signals from the sensor may lead to sensations that are useful for identification of the object and modification of ongoing movement. 

Studies of TMIS evoked Thermal Pain sensations up the burst staircase show that descriptors and pain VAS scores were constant for some sites (binary, Pain+), while at other sites they changed along the staircase (analogue, Pain−/+). The response to thalamic TMIS at Pain+ versus Pain+/− sites is consistent with the neuronal responses to painful stimuli which are either binary for thalamic NS cells and Pain+ sites, and analogue for WDR cells and pain−/+ sites [64] (Figure 2B). 

Different patterns and modalities of sensation might be implemented to optimize performance in particular situations that a patient is likely to encounter. Tactile sensations could be useful for situations in which a textured or patterned surface is scanned with minimal risk, like the use of a keyboard. Pain+ sites could be stimulated for situations requiring detection and escape from pain, while pain−/+ sites could more appropriately be stimulated for situations requiring identification and a graded response to the stimulus, such as working with utensils around a stove. TMIS evoked pain+ sensations may be useful in the case of a patient who does manual job with real risk of injury, such as a firefighter. TMIS evoked tactile, non-painful thermal and −/+ pain sensations could be combined for patients in jobs which require active touch for fine discrimination of objects and a graded response to stimuli which could be harmful, e.g., electrical wiring with a soldering gun. 

## 10. Stability of Thalamic Representations after a Major Injury 

To be effective as a locus for stimulation by signals from the sensor the somatotopy and modality of the sensation evoked by stimulation should be comparable before and after a major injury and thalamic stimulation [81,119]. In that case the patient could make use of learned experience to identify objects or carry out motor tasks. However, the results reviewed above demonstrate substantial reorganization following these injuries. For example, “Insensate” thalamic regions show increased representations of the part of the body adjacent to the area of sensory loss relative to that in “Control” and “Tremor”. 

The representation of the leg in Vc core in a patient with a Thoracic transection of the spinal cord shows no RFs while PFs were referred to the leg; above the level of the transection cutaneous stimulation numerous neurons with RFs on the chest and abdominal wall above the level of the transection (Figure 4B). The sensations evoked by TMIS were referred to the “Insensate” part of the body, which makes it likely that the activity of neurons with RFs on the chest or abdominal wall will lead to sensations in the “Insensate” part of the body. Post-inhibitory bursting in these neurons may be responsible for dysesthesias or pain in the “Insensate” parts of the body [33,36]. 

In amputees, neurons with RFs on and adjacent to the stump showed a larger thalamic representation than the corresponding part of the body in patients with ET [35,36,81]. In patients with the representation of a “phantom”, stimulation of the representation of the phantom in Vc neurons will often have RFs on the stump [85], similar to the patients with spinal transection who have persistence of the PFs in the “Insensate” lower extremities (Figure 4 (S4)). The persistence of a representation of the “Insensate” part may be related to the size of that part of the body [86]. 

The presence of a phantom is important for forebrain loci for stimulation with signals from a sensor since the patient can incorporate TMIS evoked natural sensations into established motor behaviors [84,85,120]. The approach to these cases must account for changes in the phantom representation over time and with stimulation through the electrode implanted in the thalamus [84,119]. In addition the modality of the sensation evoked by TMIS should be adjusted to reflect the distribution of receptors in the part of the body corresponding to the phantom [38,39,43,121]. 

In patients with the thalamic representation of a stump, rather than a phantom, stimulation of the stump in the periphery will produce the same sensations as stimulation of Vc, but at much lower cost and risk. In either case, the TMIS evoked PF will be referred to the stump and not to the prosthesis [84,85]. Therefore amputees must learn to incorporate unnatural sensations evoked by TMIS from sensor into established motor behaviors involving a part of the body which is no longer represented in the forebrain. This is a complex learning task like that of patients with hearing loss who must learn to use cochlear implants to hear [122,123]. 

Therefore, the use of sensors on active prostheses in a patient with a major injury will depend upon stimulation of a nervous system which has reorganized substantially. Thalamic stimulation itself will change the stimulus evoked activity of neurons in the thalamus and cortex, and the synchrony between them [119]. For these reasons, the electric stimulation paradigm must be tailored to the individual patient with an amputation including an adaptive approach to train the patient to use an active prosthesis which includes superficial somatic sensors. 

## 11. Pain Evoked by TMIS of Thalamus or Cortex

Pain is often evoked by TMIS in the Vc complex of patients with chronic pain, which is associated with a decline in the number of sites at which warm and cold (thermal) sensations is produced [82,111]. Chronic pain and hypersensitivity or evoked pain occurs in the majority of patients with spinal cord injury and amputations, in patients without and with a phantom [94,124]. TMIS evoked pains were also more common in the representation of “Insensate” parts of Vc complex of patients with amputations and spinal cord transections [36,81]. In addition, Pain+ sites defined by TMIS in patients with multiple chronic pain diagnoses were more common than in patients without chronic pain (ET). Similar observations have been reported in response to stimulation of the cortex [112,114]. 

These TMIS evoked pain sensations may limit the effectiveness of stimulation of peripheral nerves, thalamus and cortex but may be minimized by adjusting thresholds and using different patterns of stimulation (Figure 3, Pain−/+ sites) [70,107]. The ongoing chronic pain should treated by standard approaches after diagnosis into categories of neuropathic or nociceptive pain [125,126]. For example, patients with pain following spinal cord transection would be treated differently depending upon whether the pain arises within the central nervous system following spinal cord injury (Spinal Central Pain) or from nociceptors at the site of the injury to the spinal column [27,126]. The possibility that stimulation of the thalamus or motor cortex can be effective for the treatment of neuropathic and nociceptive pain should be factored into the programming of TMIS with inputs from the sensor [27,28,29]. 

Pain evoked by stimulation for forebrain loci may limit the effectiveness of TMIS of these loci with signals from sensors. TMIS often evokes pain in parts of the body where patients with major injuries experience pain, such as the stump in patients with amputation [82,111]. Many of these patients with chronic pain will also have hypersensitivity to natural or electrocutaneous stimulation of the “Insensate” part of the body in the periphery, such as the stump [125,126]. This pain and hypersensitivity must be addressed in individual patients before central or peripheral structures can be effective loci for stimulation with signals from the sensor. These factors are a challenge which may limit the use of forebrain or peripheral loci for stimulation with signals from the sensors in some patients who are candidates for an active prosthesis. 

## 12. Conclusions

There are differences in the physiology of the Vc thalamus between individual patients that may require the somatic sensory prosthesis to be tailored to the individual patient. The presence of a phantom representation in which stimulation produces natural sensations may allow the patient to adapt input from the prosthesis seamlessly into established motor activities or ‘programs’. The presence of thalamic activities, perhaps an efference copy, during imagined movements of the phantom could also facilitate this adaptation. Patients without representations of the phantom or its motor activities will require extensive training to use sensations not previously related to any movement for the conscious or unconscious control of a movement of the prosthesis. This learning or training may be similar to that required for a congenitally deaf patient to use a cochlear implant. The pain and hypersensitivity produced by TMIS at the locus for the sensory prosthesis in patients with chronic pain following a major injury must be also be tailored to the individual patient. These factors are a challenge which may limit the use of forebrain or peripheral loci for stimulation with signals from the sensors in some patients who are candidates for an active prosthesis.
